# Street working children in kurdistan region of Iraq; Mental health and traumatization

**DOI:** 10.1192/j.eurpsy.2021.1691

**Published:** 2021-08-13

**Authors:** N. Taib, H. Arinell, A. Ahmad, M. Ramklint

**Affiliations:** Neuroscience/child And Adolescent Psychiatry, Uppsala University, Uppsala, Sweden

**Keywords:** mental health, street working boys, trauma

## Abstract

**Introduction:**

Street working children are often poor, deprived of love and care, and lack supervision by responsible adults. The Kurdistan region of Iraq has experienced war conflicts for decades. Many families have been displaced and their children forced into the streets. However, little is known about mental health among the street working children in this region.

**Objectives:**

To explore mental health and trauma among street working boys in Duhok.

**Methods:**

A cross sectional study was conducted on street working boys (n=100), eight to 16 years old in Duhok. A control group of age-matched school boys (n=100) were recruited. The Child Behaviour Checklist 6-18 was used for assessment of the children’s competences and behavioural problems. Mental disorders were assessed by using the Mini-International Neuropsychiatric Interview for Children and Adolescence. Experienced trauma was assessed by the Harvard-Uppsala Trauma Questionnaire for Children.

**Results:**

Sixty-one percent of the street working boys had at least one psychiatric disorder (57 % anxiety disorders). Street working boys reported more traumatic events than school boys, 96% vs 64% (X²= 32, p < 0.001), the largest effect size was found for torture (OR 28.4) and the smallest effect size for maltreatment or assault (OR 2.7). Also, they reported higher levels of internalising symptoms, T-score 59.4 (8.2). There was a significantly increased risk of more externalising symptoms with increasing working hours, OR 2.90 [95% CI 1.02; 8.29].

**Conclusions:**

Internalizing symptoms, anxiety disorders and trauma were more common in street working boys compared to school boys. More working hours increased the risk for more externalising symptoms.

**Disclosure:**

No significant relationships.

